# Identification of potential angiogenic biomarkers in human follicular fluid for predicting oocyte maturity

**DOI:** 10.3389/fendo.2023.1173079

**Published:** 2023-08-10

**Authors:** Hsuan-Ting Chen, Wen-Bin Wu, Jun-Jin Lin, Tsung-Hsuan Lai

**Affiliations:** ^1^ Ph.D. Program in Pharmaceutic Biotechnology, Graduate Institute of Biomedical and Pharmaceutical Science, School of Medicine, Fu Jen Catholic University, New Taipei City, Taiwan; ^2^ School of Medicine, Fu Jen Catholic University, New Taipei City, Taiwan; ^3^ Assisted Reproductive Center, Department of Obstetrics and Gynecology, Cathay General Hospital, Taipei, Taiwan

**Keywords:** angiogenic factor, IVF, CXCL-6, VEGF-A, eotaxin, oocyte maturity, follicular fluid, FF

## Abstract

**Background:**

Angiogenesis in folliculogenesis contributes to oocyte developmental competence in natural and *in vitro* fertilization (IVF) cycles. Therefore, the identification of key angiogenic factors in follicular fluid (FF) during folliculogenesis is clinically significant and important for *in vitro* fertilization. This study aims to identify the key angiogenic factors in FF for predicting oocyte maturity during *in vitro* fertilization.

**Materials and methods:**

Forty participants who received ovarian stimulation using a GnRH antagonist protocol in their first *in vitro* fertilization treatment were recruited. From each patient, two follicular samples (one preovulatory follicle, > 18 mm; one mid-antral follicle, < 14 mm) were collected without flushing during oocyte retrieval. In total, 80 FF samples were collected from 40 patients. The expression profiles of angiogenesis-related proteins in FF were analyzed *via* Luminex high-performance assays. Recorded patient data included antral follicle count, anti-müllerian hormone, age, and BMI. Serum samples were collected on menstrual cycle day 2, the trigger day, and the day of oocyte retrieval. Hormone concentrations including day 2 FSH/LH/E2/P4, trigger day E2/LH/P4, and retrieval day E2/LH/P4 were measured by chemiluminescence assay.

**Results:**

Ten angiogenic factors were highly expressed in FF: eotaxin, Gro-α, IL-8, IP-10, MCP-1, MIG, PAI-1 (Serpin), VEGF-A, CXCL-6, and HGF. The concentrations of eotaxin, IL-8, MCP1, PAI-1, and VEGF-A were significantly higher in preovulatory follicles than those in mid-antral follicles, while the Gro-α and CXCL-6 expressional levels were lower in preovulatory than in mid-antral follicles (*p* < 0.05). Logistic regression and receiver operating characteristic (ROC) analysis revealed that VEGF-A, eotaxin, and CXCL-6 were the three strongest predictors of oocyte maturity. The combination of VEGF-A and CXCL-6 predicted oocyte maturity with a higher sensitivity (91.7%) and specificity (72.7%) than other combinations.

**Conclusion:**

Our findings suggest that VEGF-A, eotaxin, and CXCL-6 concentrations in FF strongly correlate with oocyte maturity from the mid-antral to preovulatory stage. The combination of VEGF-A and CXCL-6 exhibits a relatively good prediction rate of oocyte maturity during *in vitro* fertilization.

## 1 Introduction

Angiogenesis, the formation of new blood vessels from pre-existing vessels (1), is critical to ovarian follicle development and oocyte growth. The development of new blood vessels provides cytokines, growth factors, and hormones that induce follicle growth. Healthy follicles are highly vascularized, whereas those undergoing atresia have poor vascularity (2). Thus, properly functioning follicular vasculature is critically important to the fate of the follicle (3).

In the human ovary, new blood vessels form in the medulla (interior) (4) and provide nutrients by passive diffusion to the cortex (outer layer), where they induce primordial follicle development (3). Moreover, the onset of follicular vascularization begins at the early secondary stage, increases during follicular growth, and declines during follicular atresia in the marmoset (5). Thus, the decrease in vascularization is thought to be a cause or consequence of atresia, perhaps because dying follicles fail to produce angiogenic factors needed to support the vasculature. The levels of vascular endothelial growth factor (VEGF), fibroblast growth factor 2 (FGF2), growth differentiation factor-9 (GDF-9), and insulin-like growth factor (IGF) correlate with folliculogenesis and oocyte maturation (6–8). These factors are either indirectly induced or directly produced by follicular granulosa cells (GCs) and VEGF, FGF-2, and IGF have been shown to associate with angiogenesis among these factors (9, 10).

Ovarian follicular fluid (FF) contains a variety of molecules involved in oocyte maturation that are secreted by GCs, cumulus cells, and theca cells (TCs) and are transported *via* blood circulation (11). The FF includes steroid hormones, metabolites, polysaccharides, and antioxidants that provide a microenvironment for oocyte development (12, 13). Clinically, oocyte maturity (one of the factors determining oocyte quality) is commonly determined according to morphological criteria under microscopy (14). However, morphological appearance does not predict oocyte quality with absolute certainty. Therefore, more accurate tests are needed to assess oocyte quality and maturity. FF may contain molecules that could serve as biomarkers for predicting oocyte maturity and quality (15).

A prudent strategy for investigating such potential biomarkers in FF is to identify angiogenic factors essential to folliculogenesis, as angiogenesis contributes to oocyte development in the natural cycle and may also play an important role during *in vitro* fertilization (IVF) cycles. Thus, this study aims to identify the key angiogenic factor(s) in FF that are responsible for oocyte maturation during IVF.

## 2 Materials and methods

### 2.1 Patient recruitment

This study was approved by the Ethics Committee of Cathay General Hospital, Taipei, Taiwan (CGH-P107083). The study was carried out from March 2019 to March 2020, and informed consent was obtained from all patients. Patients meeting the following criteria were enrolled in the study (1): undergoing first IVF or intracytoplasmic sperm injection (ICSI) (2); age 20–45 years (3); cycle day 2 or day 3 basal follicle stimulating hormone (FSH) < 15 IU/mL (4); ovarian stimulation with gonadotropin-releasing hormone (GnRH) antagonist protocol (5); without chromosomal abnormalities. Patients with ovarian pathologies, including endometrioma, cyst (> 3cm in diameter), teratoma, and benign ovarian tumors, were excluded. A total of 40 IVF patients aged 26–44 years were enrolled. Patient clinical data collected for further evaluation included age, anti-müllerian hormone (AMH), body mass index (BMI), antral follicle counts (AFCs), basal hormone profiles, and sex hormones on human chorionic gonadotropin (hCG) day.

### 2.2 Ovarian stimulation protocol and sample collection

Patients were treated by ovarian stimulation with a GnRH antagonist protocol starting with human recombinant FSH (Follitropin Alfa, Gonal-F; Merck Serono) on menstrual cycle day 2 or 3 according to their age, BMI, and AFC. A daily subcutaneous dose of 0.25 mg of Cetrotide (Merck Serono) was started 5 to 6 days after the initiation of gonadotropins or when the mean follicle diameter was 14 mm. When two or more than two follicles were over 18 mm in diameter, the ovulation was induced using a dual trigger (hCG 6,500 U + GnRH-a 0.2 mg) (Ovidrel, Merck-Serono) (Decapeptyl, MSD). Transvaginal oocyte retrieval was performed 35–37 hours after dual trigger administration. The FF and blood serum were obtained during oocyte retrieval. Two follicular samples with one preovulatory follicle (size > 18 mm: group A) and one mid-antral follicle (follicle size < 14 mm: group B) were collected from each patient during oocyte retrieval. The maturation stage of all oocytes, including group A and group B, was recorded from patients. The oocytes were evaluated and categorized based on their nuclear maturation status into three groups: metaphase II (MII), metaphase I (MI), and germinal vesicle (GV) stages. An oocyte was categorized as metaphase I (MI) if it lacked a germinal vesicle (GV) and a polar body (PB), while an oocyte was classified as metaphase II (MII: mature oocytes) if it had a spherical shape, a uniform zona pellucida, a uniform translucent cytoplasm, and an extruded first polar body of appropriate size (16).

### 2.3 Preparation of human FF

The FF sample collection was performed as previously described by our laboratory (8). FF was collected immediately after isolation of the cumulus-oocyte complexes. The aspirates of FF containing cells such as mural GCs, erythrocytes, and leukocytes were pooled in tubes on ice. The sample collection procedure was carried out very carefully to avoid blood contamination, and the FF was obtained without washing with culture medium to minimize the wash medium volume and to avoid FF dilution. If blood contamination occurred, that FF sample was discarded. Otherwise, the FF was then further centrifuged at 1000 × *g* for 3 min at 4°C to remove any contaminating blood cells or cell debris. The FF supernatant was then aliquoted into tubes and stored at −80°C for further analysis.

### 2.4 Preparation and analysis of human serum

Patient serum samples were collected at three time points during IVF: on day 2 or 3 of the menstrual cycle, i.e. before gonadotropin administration; on the day of hCG/GnRHa administration, and 35–37 h after hCG/GnRHa administration. In contrast to the other sex hormones, the serum level of AMH (AMH Gen II assay, Beckman Coulter, Brea, CA) was measured by ELISA before the IVF cycle. All serum samples were collected into tubes and centrifuged at 1300 × *g* at 4°C for 10 min and stored at −80°C for further analyses. Serum estradiol (E2), luteinizing hormone (LH), progesterone (P4), and/or FSH levels were measured by chemiluminescence assay (Abbott Biologicals B.V., The Netherlands).

### 2.5 Luminex assay of angiogenic factors in FF

To identify angiogenic targets that were involved in folliculogenesis and oocyte maturation, 20 selected angiogenic proteins were assayed *via* Luminex. The absolute concentration of each sample was calculated from each standard. Briefly, the customized human antibody array 20-plex kit (Thermo Fisher Scientific) and ProcartaPlex antibody isotyping panel were used to perform quantitative and multiplexed protein measurements of FF using magnetic beads from Luminex. The targeted angiogenic proteins were of several categories, including 1) growth factors: HGF (hepatocyte growth factor), EGF (epidermal growth factor), FGF-2 (fibroblast growth factor-2), TGF-α (transforming growth factor-alpha), TNF-α (tumor necrosis factor-alpha), VEGF-A (vascular endothelial growth factor-A); 2) cytokines/chemokines: CXCL-6, Gro-α (growth-regulated oncogene-alpha; CXCL-1), IL-1β (interleukin-1beta), IL-6 (interleukin-6), IL-8 (interleukin-8; CXCL-8), IP-10 (interferon gamma-induced protein 10; CXCL-10), ENA-78 (epithelial-derived neutrophil-activating peptide-78; CXCL-5), eotaxin (CCL-11), IFN-γ (interferon-gamma), MCP-1 (monocyte chemoattractant protein-1), MIG (monokine induced by gamma interferon; CXCL-9), and 3) others: ANGPT1 (angiopoietin-1), MMP-2 (matrix metalloproteinase-2), PAI-1 (plasminogen activator inhibitor-1; serpin).

### 2.6 Granulosa cell culture

Follicular GCs were prepared as previously described (17). Briefly, GCs were obtained from patients undergoing oocyte retrieval for IVF. GCs in FF were isolated by centrifugation at 1000 × *g* for 3 min. The pellets with GCs were resuspended and placed in 50% Percoll solution and centrifuged at 400 × *g* for 30 min. After centrifugation, GCs retrieved from the middle of Percoll layer were cultured at M199 with 10% FBS and 100-U/mL of penicillin, 100-μg/mL streptomycin, and 25-μg/mL amphotericin B (Thermo Fisher Scientific, NY, USA) in tissue culture flasks at 37°C.

### 2.7 RT-PCR analysis

FF angiogenic factor and β-actin mRNA expression were determined by RT-PCR. The following oligonucleotide PCR primers targeting human CXCL-6, eotaxin, and β-actin were used for RT-PCR:

CXCL-6 forward primer, 5’- GGGAAGCAAGTTTGTCTGGA-3’; CXCL-6 reverse primer, 5’- CTTTCCCCCACACTCTTCAA-3’; eotaxin forward primer, 5’- CTCGCTGGGCCAGCTTCTGTC-3’; eotaxin reverse primer, 5’- GGCTTTGGAGTTGGAGATTTTTGG-3’; β-actin forward primer, 5’-ATCATGTTTGAGACCTTCAA-3’; β-actin reverse primer, 5’-CATCTCTTGCTCGAAGTCCA-3’.

The total RNA, 1st strand cDNA synthesis, PCR, and PCR product analysis were performed as previously described (18) except the annealing temperature for PCR was set at 60°C and amplification was 30 cycles.

### 2.8 Statistical analysis

Data are reported as the standard error of mean (SEM) and were compared using the paired t-test. Logistic regression and receiver operating characteristic (ROC) curve analysis were used to determine the correlation between angiogenic protein level in human FF and oocyte maturation rate. The area under the ROC curve was used to determine the probability of accurately distinguishing high-quality oocytes. Statistical significance was considered as *p* < 0.05. All analyses were performed using SPSS version 18.0 (Chicago, IL, USA).

## 3 Results

### 3.1 Patient demographics

Patient demographic data are summarized in **Supplementary Table 1**. A total of 80 FF samples including preovulatory and mid-antral follicles were collected from 40 patients with a mean age of 36.38 ± 0.79 years old, mean AMH of 3.65 ± 0.4 ng/mL, mean BMI of 20.51 ± 0.44 kg/m^2^, and mean AFC of 11.34 ± 4.53. The concentration of serum E2 on trigger day was 27.4 times higher than that on basal day and 1.75 times than that on oocyte retrieval day. The level of serum P4 on oocyte retrieval day was 29.9 and 12.2 times higher than that on basal day and trigger day, respectively.

### 3.2 Comparison of oocyte maturation rate between preovulatory and mid-antral follicles

Next, we compared oocyte maturation rate between preovulatory and mid-antral follicles. Two approaches were adopted. First, the percentage of oocyte maturity in which stage for all oocytes from the 40 patients was analyzed. Second, the fraction of oocytes in MII was compared between preovulatory (group A) and mid-antral (group B) follicles. Among them, 9 patients were undergone egg freezing without fertilization, whereas 31 patients were undergone IVF procedure. The total maturation rate of oocytes was found to be MII, 69.57%; MI, 13.87%, GV, 14.91%; and degenerative, 1.66% (**Supplementary Table 2**), in which the MII oocyte production rate is similar to that of a previous study (19). In parallel, the fraction of oocytes in MII differed significantly between group A and B, with 90.0% versus 72.5%, respectively (*p* < 0.05). The fertilization rate in group A was 80.6%, and that in group B was 67.7% (*p* > 0.05) (**Table 1**).

**Table 1:** Comparison of oocyte maturation and fertilization rate between preovulatory and mid-antral follicles.

	Group A (Preovulatory follicle; >18 mm)	Group B (Mid-antral follicle; <14 mm)	*p value*
Number of FF	40	40	
Number of oocytes	40	40	
Number of oocyte frozen	9	9	
MII (%)	36/40 (90%)	29/40 (72.5%)	0.006^*^
MI (%)	0/40 (0%)	3/40 (7.5%)	0.083
GV (%)	0/40 (0%)	7/40 (17.5%)	0.006^*^
Degeneration (%)	4/40 (10%)	1/40 (2.5%)	0.083
Number of 2PN	25	21	
Fertilization rate (%)	25/31 (80.6%)	21/31 (67.7%)	0.044^*^

FF, Follicular fluid; MII, metaphase II (mature oocyte); MI, metaphase I; GV, germinal vesicle; 2PN: 2 pronuclei (fertilized oocyte).

Fertilization rate (%): the number of fertilized oocytes/ the number of oocytes for fertilization. 9 patients underwent egg freezing only, without fertilization.

Maturation rate (%): the number of MII oocytes/ the number of total retrieved oocytes.

Statistical significance was determined by paired t-test. ^*^p < 0.05.

### 3.3 FF angiogenic protein levels differed significantly between preovulatory and mid-antral follicles

**Figure 1:**
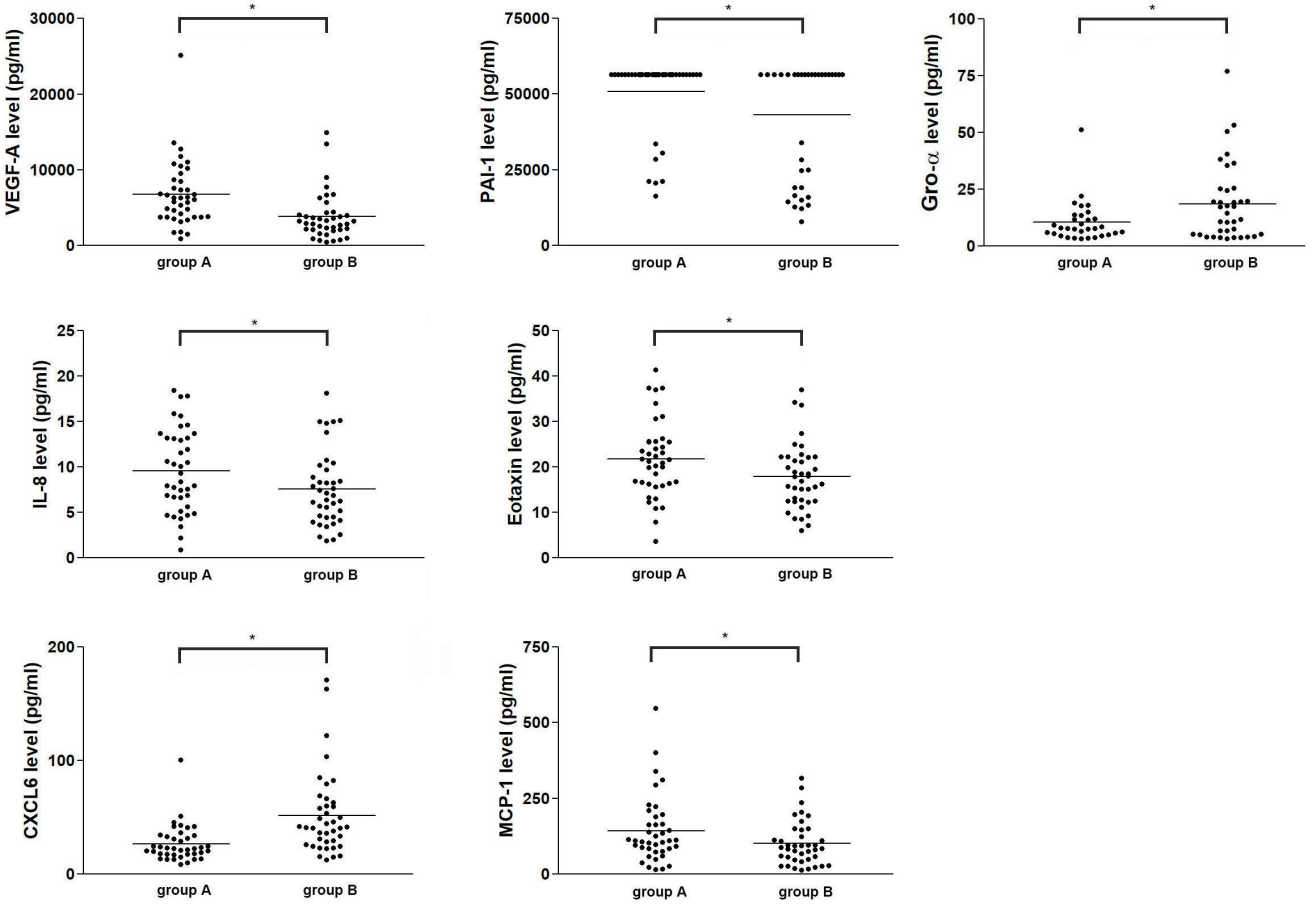
Comparison of the concentrations of seven angiogenic factors in human ovarian FF between preovulatory and mid-antral follicles. Follicles were divided into two groups according to their mean size: preovulatory follicles > 18 mm (group A) and mid-antral follicles < 14 mm (group B). The absolute concentration of an angiogenic protein in human ovarian FF was determined via Luminex assay. *p < 0.05.

To further determine whether angiogenic protein(s) in FF from preovulatory and mid-antral follicle correlate(s) with oocyte maturity, Luminex assay was performed to compare protein concentrations between FF from preovulatory and mid-antral follicles. The result showed that 10 of the targeted proteins were not detected (ND) or in a lower concentration and 3 of the targets did not differ in concentration between preovulatory and mid-antral follicles (**Supplementary Figure 1**). However, the concentrations of 7 of the angiogenic proteins in FF, including VEGF-A (*p* = 0.000), PAI-1 (*p* = 0.017), IL-8 (*p* = 0.001), MCP-1 (*p* = 0.001), eotaxin (*p* = 0.001), CXCL-6 (*p* = 0.000), and Gro-α (*p* = 0.002) differed significantly between these two groups (**Table 2** and **Figure 1**). The concentrations of eotaxin, IL-8, MCP1, PAI-1, and VEGF-A were significantly higher in preovulatory follicles than those in mid-antral follicles, while the Gro-α and CXCL-6 expressional levels were lower in preovulatory than in mid-antral follicles (*p* < 0.05). However, one may concern that some concentration data points in the PAI-1 scatter plot were near the upper limit of detection. To confirm the PAI-1 result, the original mean fluorescence intensity (MFI) data obtained from the Luminex analysis were reanalyzed. It was found that the reanalyzed result was similar to the previous one shown in **Figure 1**, demonstrating a significant higher level of PAI-1 expression in the FF of preovulatory follicle (*p* < 0.05) (**Supplementary Figure 2**). Therefore, the data presented in **Table 2** and **Figure 1** were further used in the following analyses. Logistic regression analysis revealed that the concentrations of VEGF-A, eotaxin, and CXCL-6 differed significantly between the fluid of preovulatory and mid-antral follicles (*p* < 0.05) (**Table 3**).

**Table 2:** Comparison of highly expressed angiogenic factors between preovulatory and mid-antral follicles.

Biomarkers	Group A (SEM)(Preovulatory follicle >18mm)	Group B (SEM)(Mid-antral follicle <14mm)	*p* value
FF No.	40	40	
VEGF-A (pg/mL)	6762.82 ± 686.92	3813.98 ± 492.19	0.000^*^
PAI-1 (pg/mL)	50820.15 ± 1973.13	43109.77 ± 2968.90	0.017^*^
IL-8 (pg/mL)	9.59 ± 0.72	7.55 ± 0.66	0.001^*^
MCP-1 (pg/mL)	143.13 ± 17.43	101.02 ± 11.55	0.001^*^
Eotaxin (pg/mL)	21.72 ± 1.33	17.85 ± 1.14	0.001^*^
CXCL-6 (pg/mL)	26.82 ± 2.53	51.71 ± 5.72	0.000^*^
Gro-α (pg/ml)	10.72 ± 1.68	19.97 ± 3.28	0.002^*^

Group A: preovulatory follicle >18 mm; Group B: mid-antral follicle <14 mm.

Statistical significance was determined by paired t-test. ^*^p < 0.05.

**Table 3:** Logistic regression analysis of the significant angiogenic proteins associated with oocyte maturation between preovulatory and mid-antral follicles.

Biomarkers	Odds ratio	95% C.I.	*p* value
VEGF-A	1.00061	1.00017/1.00106	0.006^*^
PAI-1	1.00004	0.99999/1.00008	0.055
IL-8	1.08495	0.91383/1.28811	0.352
MCP-1	1.00515	0.99614/1.01424	0.263
Eotaxin	1.11860	1.00812/1.24119	0.035^*^
CXCL-6	0.95568	0.91685/0.99616	0.032^*^
Gro-α	0.956	0.90462/1.01089	0.115

Statistical significance was determined using logistic regression. ^*^p < 0.05.

### 3.4 ROC analysis

**Figure 2:**
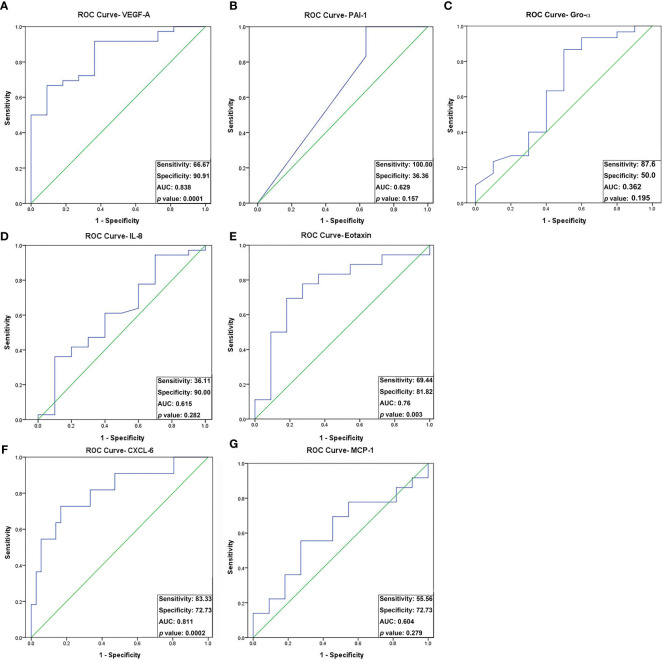
Receiver operating characteristic (ROC) curve analysis of the seven angiogenic proteins for oocyte maturity differentially expressed between preovulatory and mid-antral FF. Seven differentially expressed angiogenic factors associated with oocyte maturity were analyzed by ROC curve analysis: **(A)** VEGF-A; **(B)** PAI-1; **(C)** Gro-α; **(D)** IL-8; **(E)** Eotaxin; **(F)** CXCL-6; **(G)** MCP-1. VEGF-A, Eotaxin, and CXCL-6 concentrations differed significantly between preovulatory and mid-antral FF (*p* < 0.05). Optimal cutoff values were as follows: VEGF-A, 4521.3 pg/mL (AUC, 0.788; sensitivity, 66.7%; specificity, 90.9%); Eotaxin, 19.2 pg/mL (AUC, 0.756; sensitivity, 69.4%; specificity, 81.8%); CXCL-6, 38.5 pg/mL (AUC, 0.780; sensitivity, 83.3%; specificity, 72.7%).

The analysis of receiver operating characteristic (ROC) curves demonstrated a significant correlation between the concentrations of three angiogenic proteins, namely VEGF-A, eotaxin, and CXCL-6, and oocyte maturity (**Figure 2**). Specifically, VEGF-A and eotaxin exhibited a significantly positive correlation with oocyte maturity (AUC = 0.838, sensitivity = 66.7%, specificity = 90.9%, *p* = 0.0001 < 0.05; AUC = 0.756, sensitivity = 69.4%, specificity = 81.8%, *p* = 0.003 < 0.05; respectively) (**Figures 2A, E**). In contrast, CXCL-6 showed a significantly negative correlation with oocyte maturity (AUC = 0.811, sensitivity = 83.3%, specificity = 72.7%, *p* = 0.0002 < 0.05) (**Figure 2C**). The optimal cut-off values for VEGF-A, eotaxin, and CXCL-6 were determined as 4521.3 pg/mL, 19.2 pg/mL, and 38.5 pg/mL, respectively (**Table 4**). The detailed individual data points for these ROC curves could be found in **Supplementary Table 3**. Conversely, the concentrations of IL-8, PAI-1, Gro-α, and MCP-1 did not exhibit significant differences in relation to oocyte maturity (**Figures 2B, D, F, G**).

**Table 4:** Optimal cutoff points of VEGF-A, CXCL-6 and eotaxin level on oocyte maturation between preovulatory and mid-antral follicles.

Biomarkers	optimal cutoff values	Odds ratio	95% C.I.	*p* value
VEGF-A	4521.3 pg/mL	20.000	2.285/175.040	0.007^*^
Eotaxin	19.2 pg/mL	10.227	1.890/55.334	0.007^*^
CXCL-6	38.5 pg/mL	13.333	2.718/65.401	0.001^*^
VEGF-A with eotaxin		18.048	3.451/94.376	0.001^*^
VEGF-A with CXCL-6		35.206	3.902/317.623	0.002^*^
Eotaxin with CXCL-6		9.575	2.513/36.482	0.001^*^

Statistical significance was determined using logistic regression. ^*^p < 0.05.

**Figure 3:**
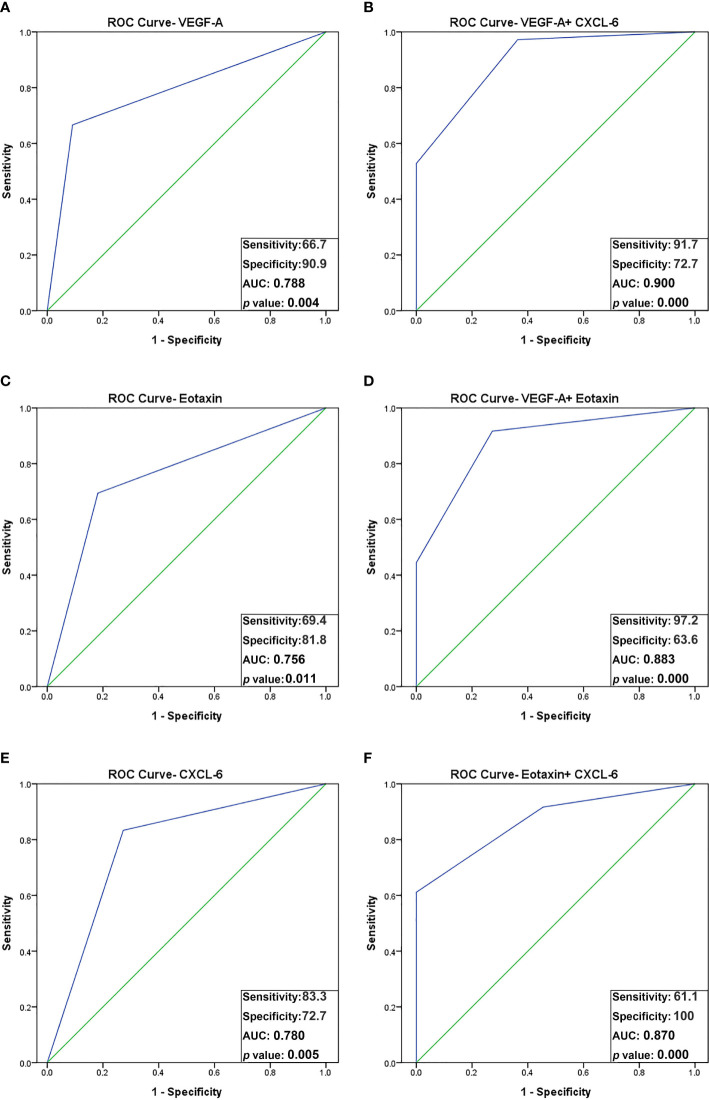
ROC curve analysis using combined biomarker signatures for predicting oocyte maturity. The ROC curves of 3 significant angiogenic proteins and the combined biomarker signatures: **(A)** VEGF-A; **(B)** VEGF-A + CXCL-6; **(C)** Eotaxin; **(D)** VEGF-A + Eotaxin; **(E)** CXCL-6; **(F)** Eotaxin + CXCL-6. The AUC for predicting oocyte maturity: VEGF-A plus CXCL-6, AUC = 0.900 (sensitivity, 91.7%; specificity, 72.7%); VEGF-A plus Eotaxin, AUC = 0.883 (sensitivity, 97.2%; specificity, 63.3%); Eotaxin plus CXCL-6, AUC = 0.870 (sensitivity, 61.1%; specificity, 100%).

The identified optimal cut-off values of VEGF-A, eotaxin, and CXCL-6 suggested that they have a good predictive power for oocyte maturity. Therefore, the ROC analysis was further conducted using these optimal cut-off values as predictors. The results revealed that VEGF-A yielded an AUC of 0.788, with a sensitivity of 66.7% and specificity of 90.9%, *p* < 0.05. Similarly, eotaxin (AUC = 0.756, sensitivity = 69.4%, specificity = 81.8%, *p* < 0.05) and CXCL-6 (AUC = 0.780, sensitivity = 83.3%, specificity = 72.7%, *p* < 0.05) exhibited considerable a predictive power for oocyte maturity (**Figures 3A-C**).

Notably, the combination of VEGF-A and CXCL-6 displayed a strikingly high AUC of 0.900, with a sensitivity of 91.7% and specificity of 72.7% in predicting oocyte maturity (*p* < 0.001). Additionally, the combination of VEGF-A and eotaxin yielded an AUC of 0.883, with a sensitivity of 97.2% and specificity of 63.6% (*p* < 0.001). Furthermore, the combination of eotaxin and CXCL-6 achieved an AUC of 0.870, with a sensitivity of 61.1% and specificity of 100% (*p* < 0.001). These findings indicate that the combination of VEGF-A and CXCL-6 outperforms all other individual factors or combinations in terms of predictive power (**Table 4** and **Figures 3D-F**).

### 3.5 CXCL-6 and eotaxin mRNA are expressed in follicular GCs

**Figure 4:**
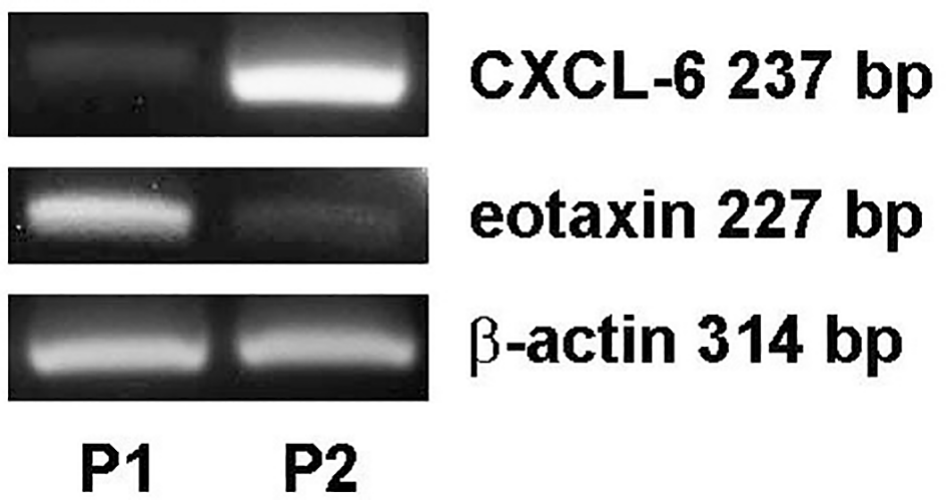
Differential expression of CXCL-6 and eotaxin mRNA in follicular GCs in patients undergoing IVF. The total mRNA was extracted from follicular GCs of two IVF patients as examples (P1: patient 1 and P2: patient 2). The differential expression of CXCL-6 and eotaxin mRNA was detected using RT-PCR.

Our previous study showed that VEGF is produced by follicular GCs and thus is likely to be present in FF (17). Because VEGF, CXCL-6, and eotaxin are three potential predictors of oocyte maturity, CXCL-6 and eotaxin mRNA expression in follicular GCs was examined by RT-PCR analysis. CXCL-6 and eotaxin mRNA expression was observed in GCs, although at different levels of expression in the two representative patients (**Figure 4**). These results suggest that follicular GCs may be a secretory source of the CXCL-6 and eotaxin found in FF.

## 4 Discussion

In this study, we identified 7 angiogenesis-related proteins that were differentially expressed between preovulatory and mid-antral FF (**Figure 1**). Of these, VEGF-A, eotaxin, and CXCL-6 concentrations strongly correlated with oocyte maturity. The correlation with oocyte maturation was positive for VEGF-A and eotaxin, but negative for CXCL-6 (**Figure 1**, **2**). Two of the three significantly-expressed angiogenic factors (eotaxin and CXCL-6) belong to the chemokine family (20). Previous studies have shown that VEGF and eotaxin play roles in angiogenesis (17, 21). Additionally, VEGF is one of the most relevant angiogenic factors studied to date in orchestrating folliculogenesis (22). Elevated VEGF levels in FF have been observed in both natural and IVF cycles, and it has been established that mature follicles originate from highly vascularized follicles (23, 24). Moreover, in hormone-stimulated IVF cycles involving patients with normal ovarian response, a positive correlation was found between VEGF levels in FF and the extent of peri-follicular vascularity on the day of follicle aspiration (25). Nevertheless, our current study revealed that the area under the curve (AUC) of VEGF in predicting oocyte maturity was only 0.788 (**Figure 3**), indicating that FF VEGF alone is insufficient to serve as a robust predictor of oocyte maturity.

Follicles with a diameter of 16–22 mm on trigger day are most likely to contain mature oocytes in IVF (26). Therefore, the follicle size appears to indicate the timing of the final follicular maturation trigger (27). In addition, healthy follicles are highly vascularized, suggesting a relationship between follicular vascularization and follicular function (28). Predicting oocyte maturity using follicle size is not a perfect method as it might cause an interpersonal and intrapersonal errors of measurement. Therefore, measuring the serum concentration of estradiol is an alternative method for predicting oocyte maturity (200 pg/mL per mature oocyte). In this study, we observed that follicles larger than 18 mm in diameter (preovulatory follicle) had an oocyte maturation rate of 90%, whereas those smaller than 14 mm (mid-antral follicle) had a maturation rate up to 72.5% (**Table 1**). Thus, follicle size neither can perfectly predict oocyte maturity nor follicular maturity. Two related limitations are encountered in the clinical practice. First, identifying the stage of oocyte maturation prior to cumulus cell removal presents challenges. Second, inducing oocyte maturation *via* artificial methods after removing cumulus cells is time-consuming. Therefore, the identification of key angiogenic factor(s) in FF that are responsible for oocyte maturation during IVF could lead to new methods for determining oocyte maturity.

Eotaxin (also known as CCL11), an 8.3-kDa protein belonging to the chemokine CC family, interacts with eosinophils (EOS) through CC chemokine receptor 3 (CCR3) (29). EOS preferentially accumulates in dilated microvessels of the thecal layer transforming into septae of the corpus luteum. The number of extravasated EOS was observed to be low in the granulosa layer under luteinization, moderate in the thecal layer, and high in hemorrhages in the former antrum (30). Eotaxin binds to human endothelial cells to induce endothelial proliferation and migration (31, 32). Elevated eotaxin expression in FF might play an important role in angiogenesis and oocyte maturation during the stage of ovarian angiogenesis between the LH surge and meiosis completion (33, 34). This phenomenon would explain the higher eotaxin level observed in the preovulatory follicle during IVF.

We found that the expression level of CXCL-6 was higher in mid-antral FF than that in preovulatory FF, demonstrating its negative correlation with oocyte maturity. A previous study showed that CXCL-6 is a neutrophil-activating chemokine with bactericidal properties (35). Torán et al. reported that CXCL-6 is an important paracrine factor in the pro-angiogenic human cardiac progenitor-like cell secretome (36). Further, VEGF-A, IGF-1, HGF, and IL-8 gene expression was promoted at a high level by CXCL-6 in a myocardial infarction model (37). Hence, the higher level of CXCL-6 in small follicles (mid-antral follicles; group B) implies that CXCL-6 may affect oocyte maturity in an earlier phase of follicle development. On the contrary, VEGF-A may influence oocyte maturity in late phase (preovulatory phase; group A). Whether the two factors work together or independently to contribute to oocyte maturation remains to be determined. However, our study, at least in part, revealed that the combination of CXCL-6 and VEGF can be a better predictor for oocyte maturation in IVF.

To our knowledge, few studies have investigated the relationship between follicular angiogenic factors and oocyte maturation in preovulatory and mid-antral FF in the human ovary. FF is a plasma filtrate with a large dynamic range of protein concentrations that render the detection of lower-abundance proteins challenging (38). Luminex multiplex assays are designed to simultaneously detect and quantitate multiple secretory proteins (e.g., cytokines, chemokines, and growth factors) with greater efficiency and high throughput. Our results may pave the way for the selection of angiogenic factors in FF for use in predicting oocyte maturity. However, the following limitations of this study should be addressed: 1) this study was a prospective and observational study, and the results should be further validated by a well-designed randomized controlled trial; 2) the relatively small sample size may produce bias in statistical data; 3) due to limited FF sample amount, the absolute PAI-1 concentrations of some patients near upper limits cannot be reanalyzed by Luminex assay. Therefore, the non-involvement of PAI-I as a potential angiogenic factor doesn’t mean to exclude the possibility of its potential. Inversely, based on our analysis on the original MFI data (**Supplementary Figure 2**), PAI-1 may also be a potential factor for predicting oocyte maturation, although it needs to be further investigated; 4) the mechanisms underlying the action of FF angiogenic factors on oocyte maturation were not investigated in this study. Further studies in cultured GCs and animal models are needed to address this question.

In this study, we did not include parameters such as age, BMI, AMH, AFCs, and hormones for statistical analyses in the two groups. The main focus of this study was to compare the differences in follicular angiogenic factors between preovulatory and mid-antral follicles from the same patient. Therefore, we collected two follicular samples, one preovulatory follicle (size > 18 mm: group A) and one mid-antral follicle (size < 14 mm: group B), during oocyte retrieval. Consequently, there is no need to adjust the results based on each patient’s age, BMI, AMH, AFCs, and hormones. Hence, we utilized the paired t-test to compare the differences in follicular angiogenic factors between these two groups. The mean age of the study participants was 36.38 ± 0.79 years. Each patient contributed two follicular samples, one from group A and one from group B, ensuring that the follicular fluid (FF) samples were obtained from the same patient. Consequently, any age-related effects should be offset between the two groups. However, the impact of age on the expression of angiogenic factors could not be assessed due to the limited sample size of only 40 patients. We intend to investigate this aspect in future studies, where a larger sample size can provide more robust insights into the relationship between age and angiogenic factor expression.

In conclusion, FF VEGF-A, eotaxin, and CXCL-6 are involved in oocyte maturation during the mid-antral to preovulatory stage. These three angiogenic factors can be used individually as a biomarker to predict oocyte maturity. In addition, a combination of CXCL-6 and VEGF can be a better predictor for oocyte maturity in IVF, revealing a potential application of FF CXCL-6 and VEGF in judging oocyte maturity during IVF. In this regard, it is possible to develop a protein assay kit for fast predicting oocyte maturity by targeting these biomarkers. However, further efforts are required to explore the actual biological roles of the three angiogenic factors and combined impact of VEGF-A and CXCL-6 in oocyte maturity prediction in folliculogenesis in IVF cycles.

## Data availability statement

The original contributions presented in the study are included in the article/**Supplementary Material**. Further inquiries can be directed to the corresponding author.

## Ethics statement

The Ethics Committee of Cathay General Hospital approved this study (IRB no.: CGH- P107083), and written informed consent was obtained from all patients. The patients/participants provided their written informed consent to participate in this study.

## Author contributions

H-TC drafted the manuscript. H-TC, W-BW, and T-HL conceived and designed the study. W-BW, H-TC, and J-JL contributed to the statistical analysis and interpretation of data. T-HL participated in the discussion and critically revised the manuscript. All authors contributed to the article and approved the submitted version.
