# Supplementary material for: Identification of potential angiogenic biomarkers in human follicular fluid for predicting oocyte maturity
Source: Front Endocrinol (Lausanne). 2023 Aug 10;14:1173079. doi: 10.3389/fendo.2023.1173079 (PMC10448508; doi:10.3389/fendo.2023.1173079)
Supplement: Supplementary file 1 [file DataSheet_1.docx]

Supplementary Materials and Results

**Supplementary Table 1. Clinical characteristics of patients**

|  | **Mean ± SE** | **Median (IQR)** |
| --- | --- | --- |
| Patient No.  Age (years) | 40  36.38 ± 0.79 | 40  36.50 |
| AMH (ng/mL) | 3.65 ± 0.40 | 2.93 |
| BMI (kg/m^2^) | 20.51 ± 0.44 | 20.00 |
| AFCs | 11.34 ± 4.53 | 10.00 |
| Basal serum E2 (pg/mL) | 144.50 ± 110.46 | 33.14 |
| Basal serum LH (mIU/mL) | 6.31 ± 0.53 | 6.17 |
| Basal serum P4 (pg/mL) | 0.54 ± 0.18 | 0.28 |
| Basal serum FSH (mIU/mL) | 7.49 ± 0.36 | 7.46 |
| Serum E2 (pg/mL) on trigger day | 3954.56 ± 540.67^*^ | .2759.50 |
| Serum LH (mIU/mL) on trigger day | 2.22 ± 0.28^*^ | 1.78 |
| Serum P4 (pg/mL) on trigger day | 1.32 ± 0.21^*^ | 0.90 |
| Serum E2 (pg/mL) on oocyte retrieval day | 2260.22 ± 376.90^#^ | 1772.00 |
| Serum LH (mIU/mL) on oocyte retrieval day | 4.67 ± 0.70^#^ | 3.49 |
| Serum P4 (pg/mL) on oocyte retrieval day | 16.14 ± 2.97^#^ | 11.96 |

AMH, anti-müllerian hormone; AFCs, antral follicle counts; E2, estradiol; P4, progesterone; LH, luteinizing hormone; FSH, follicle-stimulating hormone. ^*^serum sex hormone/gonadotropin level on day 2 or day 3 (basal day) compared with that on trigger day; ^#^ serum sex hormone/gonadotropin level on trigger day compared to that on oocyte retrieval day; ^*^*p* < 0.05

**Supplementary Table 2. Fraction of retrieved oocytes in each stage of development**

| **Oocyte stage** | **Number** | **Fraction (%)*** |
| --- | --- | --- |
| MII | 336 | 69.57 |
| MI | 67 | 13.87 |
| GV | 72 | 14.91 |
| Degeneration | 8 | 1.66 |

*Total number of oocytes retrieved from 40 patients = 483. MII, metaphase II; MII, metaphase I; GV, germinal vesicle

**Supplementary Table 3.** ROC curves- all data points of VEGF-A.

| **Coordinates of the Curve**  Test Result Variable(s): VEGF-A | | |  |
| --- | --- | --- | --- |
| Positive if Greater Than or Equal Toa | Sensitivity | 1 - Specificity |  |
| 436.9100 | 1.000 | 1.000 |  |
| 598.7200 | 1.000 | .909 |  |
| 823.6000 | 1.000 | .818 |  |
| 895.8300 | .972 | .818 |  |
| 1210.7600 | .972 | .727 |  |
| 1673.2300 | .944 | .727 |  |
| 1887.8500 | .917 | .727 |  |
| 2194.2500 | .917 | .636 |  |
| 2570.6900 | .917 | .545 |  |
| 2836.0900 | .917 | .455 |  |
| 3050.1000 | .917 | .364 |  |
| 3273.3050 | .889 | .364 |  |
| 3466.0750 | .861 | .364 |  |
| 3628.3850 | .833 | .364 |  |
| 3748.2250 | .806 | .364 |  |
| 3762.2600 | .778 | .364 |  |
| 3773.0150 | .750 | .364 |  |
| 3796.0950 | .722 | .364 |  |
| 3825.0800 | .722 | .273 |  |
| 3961.5900 | .694 | .273 |  |
| 4160.8350 | .694 | .182 |  |
| 4310.0950 | .667 | .182 |  |
| **4521.2650** | **.667** | **.091** |  |
| 4727.5300 | .639 | .091 |  |
| 4832.5300 | .611 | .091 |  |
| 5276.4750 | .583 | .091 |  |
| 5738.7050 | .556 | .091 |  |
| 5925.7400 | .528 | .091 |  |
| 6192.2850 | .500 | .091 |  |
| 6323.8450 | .500 | .000 |  |
| 6337.3050 | .472 | .000 |  |

| 6549.3000 | .417 | .000 |
| --- | --- | --- |
| 6778.4400 | .389 | .000 |
| 7105.3950 | .361 | .000 |
| 7355.5100 | .333 | .000 |
| 7482.7350 | .306 | .000 |
| 8025.6450 | .278 | .000 |
| 8590.3700 | .250 | .000 |
| 9463.0750 | .222 | .000 |
| 10336.3950 | .194 | .000 |
| 10635.9550 | .167 | .000 |
| 10922.7400 | .139 | .000 |
| 11431.5300 | .111 | .000 |
| 12284.6850 | .083 | .000 |
| 13172.6300 | .056 | .000 |
| 19356.8550 | .028 | .000 |
| 25125.1300 | .000 | .000 |

**Supplementary Table 3.** ROC curves- all data points of Eotaxin.

| **Coordinates of the Curve**  Test Result Variable(s): Eotaxin | | |
| --- | --- | --- |
| Positive if Greater Than or Equal Toa | Sensitivity | 1 - Specificity |
| 2.6300 | 1.000 | 1.000 |
| 5.7350 | .972 | 1.000 |
| 8.2350 | .944 | 1.000 |
| 8.9500 | .944 | .909 |
| 9.5650 | .944 | .818 |
| 10.4000 | .944 | .727 |
| 10.9650 | .917 | .727 |
| 11.0600 | .889 | .727 |
| 11.7850 | .889 | .636 |
| 12.7250 | .889 | .545 |
| 13.1300 | .861 | .545 |
| 14.3200 | .833 | .545 |
| 15.5100 | .833 | .455 |
| 15.6450 | .833 | .364 |
| 15.7700 | .806 | .364 |
| 16.0450 | .778 | .364 |
| 16.4050 | .778 | .273 |
| 16.7300 | .750 | .273 |
| 17.7100 | .722 | .273 |
| 18.5650 | .694 | .273 |
| **19.2300** | **.694** | **.182** |
| 19.9350 | .667 | .182 |
| 20.1300 | .639 | .182 |
| 20.5800 | .611 | .182 |
| 21.0850 | .583 | .182 |
| 21.4500 | .556 | .182 |
| 21.6650 | .528 | .182 |
| 21.9100 | .500 | .182 |
| 22.2450 | .500 | .091 |
| 22.6650 | .472 | .091 |
| 23.0150 | .444 | .091 |

| 23.7900 | .389 | .091 |
| --- | --- | --- |
| 24.2200 | .361 | .091 |
| 24.9200 | .333 | .091 |
| 25.4650 | .306 | .091 |
| 25.5400 | .278 | .091 |
| 25.6350 | .250 | .091 |
| 25.9650 | .222 | .091 |
| 28.4600 | .194 | .091 |
| 30.9250 | .167 | .091 |
| 32.6400 | .139 | .091 |
| 34.1800 | .111 | .091 |
| 35.6600 | .111 | .000 |
| 37.2200 | .083 | .000 |
| 37.4150 | .056 | .000 |
| 39.4400 | .028 | .000 |
| 42.4500 | .000 | .000 |

**Supplementary Table 3.** ROC curves- all data points of CXCL-6.

| **Coordinates of the Curve** | | |
| --- | --- | --- |
| Test Result Variable(s): CXCL-6 | | |
| Positive if Less Than or Equal Toa | Sensitivity | 1 - Specificity |
| 7.7900 | .000 | .000 |
| 9.3450 | .028 | .000 |
| 11.4200 | .056 | .000 |
| 13.0650 | .083 | .000 |
| 13.3050 | .111 | .000 |
| 13.5750 | .139 | .000 |
| 14.3050 | .167 | .000 |
| 15.0150 | .194 | .000 |
| 16.0400 | .194 | .091 |
| 17.2100 | .222 | .091 |
| 17.6450 | .250 | .091 |
| 18.0500 | .278 | .091 |
| 18.6850 | .306 | .091 |
| 19.5450 | .333 | .091 |
| 20.3000 | .361 | .091 |
| 20.6150 | .389 | .091 |
| 20.9750 | .417 | .091 |
| 21.4250 | .444 | .091 |
| 22.0200 | .472 | .091 |
| 22.5550 | .500 | .091 |
| 22.7650 | .528 | .091 |
| 23.1050 | .528 | .182 |
| 23.5050 | .556 | .182 |
| 24.1700 | .583 | .182 |
| 24.6900 | .611 | .182 |
| 27.0000 | .639 | .182 |
| 29.3850 | .667 | .182 |
| 30.2200 | .667 | .273 |
| 31.2950 | .694 | .273 |
| 32.3250 | .722 | .273 |
| 33.6300 | .750 | .273 |
| 35.5150 | .806 | .273 |
| **38.5100** | **.833** | **.273** |
| 40.7950 | .833 | .364 |
| 41.3450 | .861 | .364 |
| 41.7650 | .861 | .455 |
| 42.4650 | .889 | .455 |
| 44.2450 | .917 | .455 |
| 47.3400 | .944 | .455 |
| 49.6500 | .944 | .545 |
| 50.5950 | .944 | .636 |
| 54.4350 | .972 | .636 |
| 63.4650 | .972 | .727 |
| 84.8000 | .972 | .818 |
| 131.9650 | 1.000 | .818 |
| 167.1050 | 1.000 | .909 |
| 171.9000 | 1.000 | 1.000 |

**
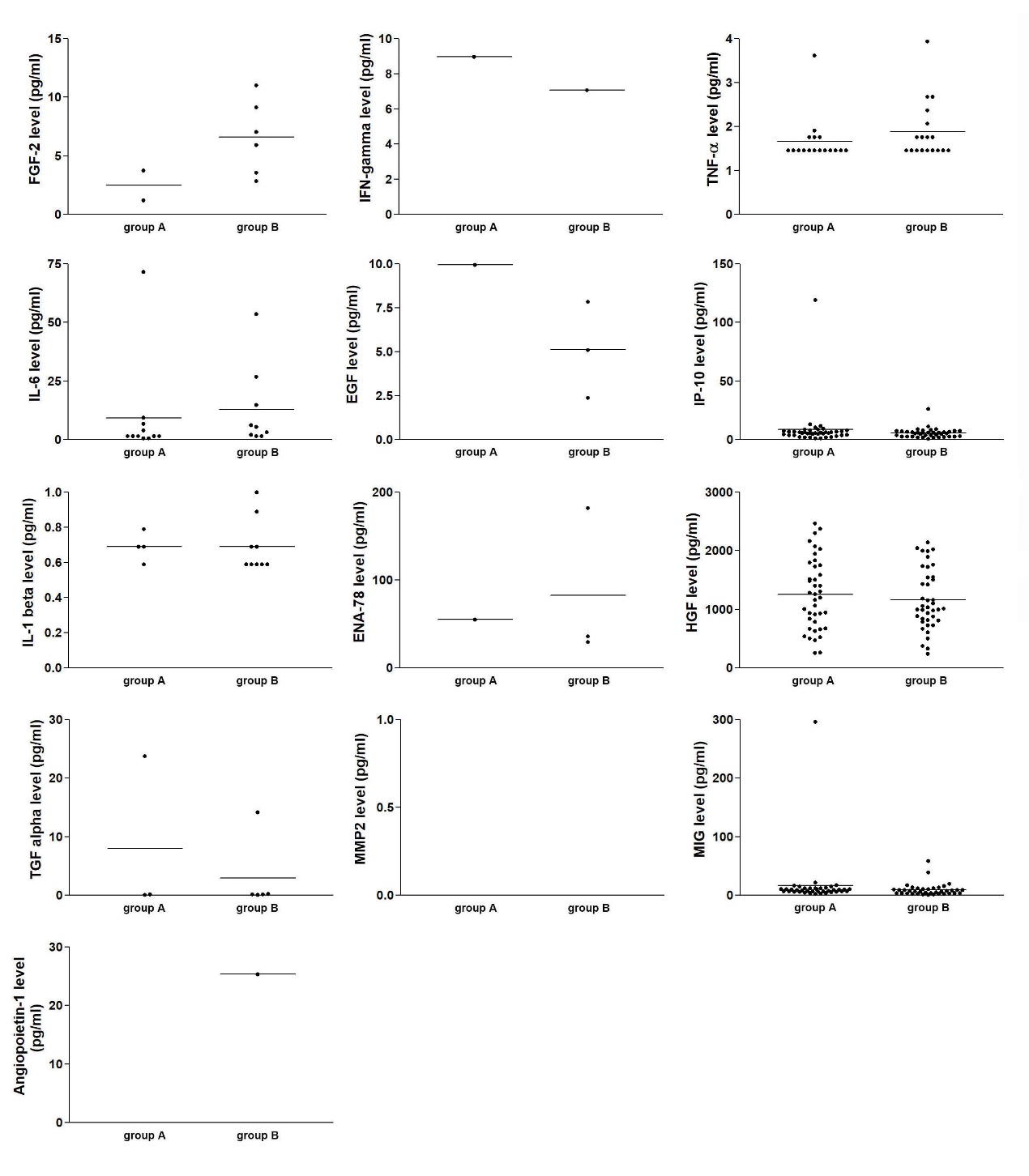
**

**Supplementary Figure 1. Expression profile of nonsignificant angiogenic proteins in human ovarian FF from preovulatory and mid-antral follicles.** Follicles were divided into two follicular groups according to their maximal dimensional size: preovulatory follicles, > 18 mm (group A); mid-antral follicles, < 14 mm (group B).


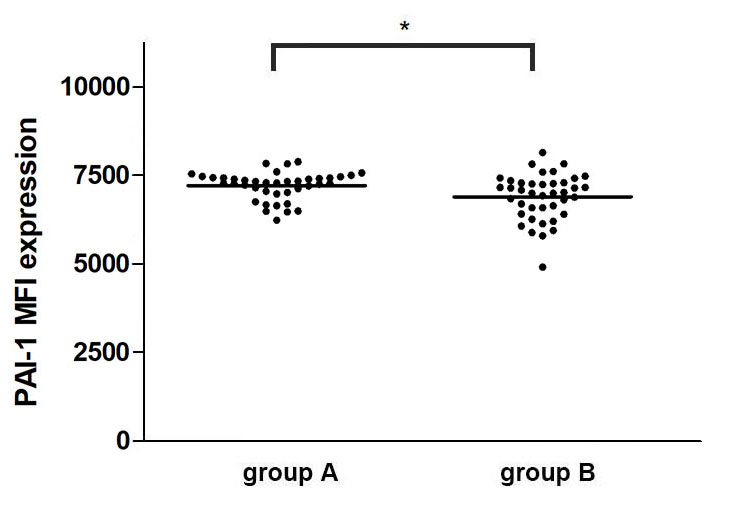


**Supplementary Figure 2 Comparison of the mean fluorescence intensity (MFI) of PAI-1 in human ovarian FF between preovulatory and mid-antral follicles.** Follicles were divided into two groups according to their mean size: preovulatory follicles > 18 mm (group A) and mid-antral follicles < 14 mm (group B). ^*^*p* < 0.05
